# The attitude and practice of general surgeons toward cytoreductive surgery and hyperthermic intraperitoneal chemotherapy: A cross-sectional study

**DOI:** 10.1016/j.amsu.2021.102440

**Published:** 2021-06-03

**Authors:** Thamer A. Bin Traiki, Sulaiman A. AlShammari, Wadha S. AlOtaibi, Shahad N. AlAnazi, Mashal M. Alnmry, Abdullah M. Albdah, Noura S. Alhassan

**Affiliations:** a Colorectal Research Chair, Department of Surgery, College of Medicine, King Khalid University Hospital, King Saud University, Riyadh, 11461, Saudi Arabia; b Trauma and Acute Care Surgery Fellow, Department of Surgery, King Saud University Medical City, King Saud University, Riyadh, 11461, Saudi Arabia

**Keywords:** Cytoreductive surgery (CRS), Hyperthermic intraperitoneal chemotherapy (HIPEC), Peritoneal carcinomatosis (PC), General surgery, Oncology

## Abstract

**Background:**

Cytoreductive surgery (CRS) and hyperthermic intraperitoneal chemotherapy (HIPEC) mandate well-established HIPEC and oncology centers, which are not available in many medical institutions. This study assessed the knowledge, attitude, and practice toward CRS and HIPEC of general surgeons in Riyadh, Saudi Arabia.

**Patients and methods:**

General surgeons (n = 266) from nine hospitals who treat patients with gastrointestinal cancer were surveyed. The responses of surgeons who work in HIPEC and academic centers (Group A) and surgeons working in tertiary and secondary hospitals (Group B) were compared. The survey response rate was 48.1% (128/266).

**Results:**

Surgeons in group B treated significantly more patients with peritoneal carcinomatosis per year than surgeons in group A (*P* = .001). Group B reported having a HIPEC specialist at their hospital, and 71.4% reported that the nearest HIPEC center was within 30 miles, compared to 4.5% of respondents in group A (*P* = .001). Lack of access to a HIPEC specialist was reported by 15.5% of surgeons in group B and 0% of surgeons in group A (*P* = .006). HIPEC as a possible therapeutic option for appendiceal cancer was cited by 60.7% of surgeons in group B compared to 84.1% of surgeons in group A (*P* = .007) and as a therapeutic option for ovarian cancer by 52.4% of surgeons in group B and 81.8% of surgeons in group A (*P* = .001).

**Conclusion:**

New strategies are needed to improve the knowledge and implementation of the referral system for HIPEC among general surgeons. Our study was limited by a low response rate.

## Highlights

● Exposure to HIPEC centers during residency or fellowship training was reported by half of our respondents.
● Surgeons in tertiary and secondary hospitals treated significantly more patients with peritoneal carcinomatosis per year than surgeons in HIPEC and academic centers.
● Lack of access to a HIPEC specialist was significantly reported by surgeons working in tertiary and secondary hospitals.
● HIPEC was significantly cited as an option for appendiceal cancer by surgeons in HIPEC and academic centers compared to surgeons in tertiary and secondary hospitals.

## 1 Introduction

Peritoneal carcinomatosis (PC) is associated with cancers of the gastrointestinal (GI), reproductive, and genitourinary tracts. However, the most common associated cancers are ovarian, colon, and gastric [1]. PC is associated with a poor prognosis and was once believed to be an incurable component of intra-abdominal malignancies, as systemic chemotherapy resulted in no long-term survival and poor quality of life in patients with PC [1,2]. The development of cytoreductive surgery (CRS) and hyperthermic intraperitoneal chemotherapy (HIPEC) significantly improved the prognosis of patients with PC, originating from most GI and genitourinary carcinomas [1,3,4]. A recent trial reported that CRS and HIPEC for PC Results in 5-year disease-free survival rates of more than 30% of patients [5].

A lack of referrals to HIPEC experts and the underutilization of both CRS and HIPEC limit the success of these therapies and are due to several factors, including limited access to HIPEC experts, a perception of insufficient evidence for these therapeutic methods, and a lack of familiarity with the data regarding the outcomes, impacts, referral patterns, and management choices [6]. As multidisciplinary teamwork has been proven to improve the diagnostic accuracy and overall survival of patients with cancer, raising awareness among general surgeons regarding the appropriate utilization of CRS and HIPEC is essential [7]. An assessment of general surgeons’ knowledge of HIPEC and PC is necessary to ensure appropriate treatment by highlighting the need for educational and training programs [8]. This study assessed the knowledge and experience regarding CRS and HIPEC for PC of general surgeons in Riyadh, Saudi Arabia.

### 1.1 Patients and Methods

This study was approved by the institutional review board committee at King Saud University Medical City. The general surgeons from nine hospitals were included in the study after applying a multistage sampling that started with a stratified sampling. The strata were composed of HIPEC centers, major tertiary and academic hospitals, and secondary hospitals. A representative, proportional sample from each stratum was used to form clusters that were proportional to the hospitals in each stratum: three HIPEC centers, three major tertiary and academic hospitals, and three secondary hospitals. All participants who were exposed to patients with gastrointestinal cancer were included in the study. We collected data regarding the surgeons’ demographics and background, attitude, and knowledge regarding CRS and HIPEC. Surgeons were grouped by their work environments: surgeons working at HIPEC and academic centers were allotted to group A, while group B consisted of surgeons working at tertiary and secondary hospitals.

### 1.2 Data collection

Information regarding the study and the questionnaire was provided to all participants, and written informed consent was obtained. Both the questionnaire and the consent form were completed online using Google Forms [9]. This work has been reported in line with the STROCSS [10].

### 1.3 Survey questionnaire

A previously validated and published questionnaire [6] was modified by an expert oncology surgeon and a researcher using feedback from a small group of general surgeons for use in this study. The questionnaire included items regarding demographic information, practice and experience, the availability of HIPEC centers and specialists, factors that influenced referral decisions, and general knowledge regarding CRS and HIPEC.

### 1.4 Statistical analysis

Statistical Package for Social Sciences version 23.0 software (SPSS Inc, IBM, Armonk, New York, USA) was used for the statistical analyses in this study. The Results are expressed as numbers and percentages. The chi-square test was used to determine the significant differences between groups A and B. Statistical significance was set at *P* < .05.

## 2 Results

A total of 266 surgeons from nine hospitals in Riyadh were surveyed, and 128 responded (response rate: 48.1%). Overall, 98 respondents (76.6%) were male, and 44 were allocated to group A, while 84 were allocated to group B. Half of the respondents (65; 50.8%) identified as registrar or specialist surgeons, 46 (35.9%) identified as consultants, and 17 (13.3%) identified as fellows. A total of 97 respondents (75.8%) reported having > 5 years of experience practicing medicine. While 68 respondents (53.1%) identified as general surgeons, 60 (46.9%) specialized in acute care, breast and endocrine, colorectal, hepatobiliary, oncologic, or upper GI surgery. The respondents’ characteristics are summarized in Table 1.

**Table 1:** Respondent characteristics.

	All respondents N = 128	Group A N = 44	Group B N = 84	*P*
Sex				
Male	98 (76.6%)	31 (70.5%)	67 (79.8%)	.238
Female	30 (23.4%)	13 (29.5%)	17 (20.2%)	
Surgical Specialty				
Acute care	8 (6.3%)	6 (13.6%)	2 (2.4%)	
Breast and endocrine	6 (4.7%)	4 (9.1%)	2 (2.4%)	
Colorectal	16 (12.5%)	9 (20.5%)	7 (8.3%)	
General	68 (53.1%)	10 (22.7%)	58 (69.0%)	<.001
Hepatobiliary	5 (3.9%)	5 (11.4%)	0	
Surgical oncology	4 (3.1%)	2 (4.5%)	2 (2.4%)	
Upper GI	12 (9.4%)	5 (11.5%)	7 (8.3%)	
Upper GI + General	5 (3.9%)	1 (2.3%)	4 (4.8%)	
Other	4 (3.1%)	2 (4.5%)	2 (2.4%)	
Level				
Consultant	46 (35.9%)	26 (59.1%)	20 (23.8%)	<.001
Fellow	17 (13.3%)	9 (20.5%)	8 (9.5%)	
Registrar/Specialist	65 (50.8%)	9 (20.5%)	56 (66.7%)	
Years in practice				
≤5 years	31 (24.2%)	17 (38.6%)	14 (16.7%)	.006
>5 years	97 (75.8%)	27 (61.4%)	70 (83.3%)	

Abbreviations: GI- gastrointestinal.

Exposure to HIPEC centers during residency or fellowship training was reported by 54.5% of respondents in group A and 51.2% in group B (Table 2). A higher proportion of respondents in group B (41.7%) learned about CRS and HIPEC during training than in group A (31.8%). Most respondents (90.9% of group A and 91.7% of group B) reported having treated patients with GI cancers. The discussion of the management of those patients in a multidisciplinary tumor board meeting was reported by 77.3% of respondents in group A and 50% in group B. A higher proportion of respondents in group B (84.5%) reported treating at least 15 patients with GI cancer and peritoneal metastasis per year than in group A (50.0%) (*P* = .001). However, fewer respondents in group B reported having a HIPEC specialist in their center (42 (95.5%) in group A and 25 (29.8%) in group B). Only six respondents (7.1%) in group B reported that the nearest HIPEC center was at the same hospital, while 40 respondents (90.9%) in group A reported having a HIPEC center at the same hospital. The nearest HIPEC center was reported as within 30 miles by 71.4% of respondents in group B and 4.5% in group A and over 30 miles away by 21.4% of respondents in group B and 4.5% in group A (*P* = .001) (see Table 3).

**Table 2:** Respondents’ exposure to CRS and HIPEC.

	All Respondents	Group A	Group B	*P*
	N = 128	N = 44	N = 84	
Were you exposed to a HIPEC center during residency or fellowship training?				
Yes	67 (52.3%)	24 (54.5%)	43 (51.2%)	.718
No	61 (47.7%)	20 (45.5%)	41 (48.8%)	
Where did you learn about CRS and HIPEC?				
Following cancer patient	1 (0.8%)	0	1 (1.2%)	
From colleague	28 (21.9%)	12 (27.3%)	16 (19.0%)	
Peer-reviewed literature	13 (10.2%)	2 (4.5%)	11 (13.1%)	.171
Colleague + peer-reviewed	9 (7.0%)	5 (11.4%)	4 (4.8%)	
Training programs	49 (38.3%)	14 (31.8%)	35 (41.7%)	
Training program + colleagues	12 (9.4%)	7 (15.9%)	5 (6.0%)	
Training program + peer-reviewed	2 (1.6%)	0	2 (2.4%)	
Training + peer review + colleagues	14 (10.9%)	4 (9.1%)	10 (11.9%)	
Treated patients with GI cancer?				
Yes	117 (91.4%)	40 (90.9%)	77 (91.7%)	.885
No	11 (8.6%)	4 (9.1%)	7 (8.3%)	
Number of patients with peritoneal metastases from GI cancers seen in a year				
Never	11 (8.6%)	6 (13.6%)	5 (6.0%)	<.001
<5	62 (48.4%)	14 (31.8%)	48 (57.1%)	
5–15	31 (24.2%)	8 (18.2%)	23 (27.4%)	
>15	24 (18.8%)	16 (36.4%)	8 (9.5%)	
How often is management discussed at the multidisciplinary tumor board?				
Never	19 (14.8%)	6 (13.6%)	13 (15.5%)	.006
Rarely	33 (25.8%)	4 (9.1%)	29 (34.5%)	
About half of the time	5 (3.9%)	1 (2.3%)	4 (4.8%)	
Most of the time	71 (55.5%)	33 (75.0%)	38 (45.2%)	
Is there a surgeon with expertise in CRS and HIPEC available at your hospital?				
Yes	67 (52.3%)	42 (95.5%)	25 (29.8%)	<.001
No	61 (47.7%)	2 (4.5%)	59 (70.2%)	
The closest HIPEC center available is				
At the same hospital	46 (35.9%)	40 (90.9%)	6 (7.1%)	<.001
<30 miles away	62 (48.4%)	2 (4.5%)	60 (71.4%)	
>30 miles away	20 (15.6%)	2 (4.5%)	18 (21.4%)	

Abbreviations: CRS- cytoreductive surgery; HIPEC- hyperthermic intraperitoneal chemotherapy; GI- gastrointestinal.

**Table 3:** Respondents’ attitude and knowledge regarding CRS and HIPEC.

	All Respondents	Group A	Group B	*P*
	N = 128	N = 44	N = 84	
Have you ever referred a patient to an HIPEC specialist for CRS and HIPEC?				
Yes	90 (70.3%)	31 (70.5%)	59 (70.2%)	.980
No	38 (29.7%)	13 (29.5%)	25 (29.8%)	
Select all of the reasons why you have not referred a patient to a HIPEC specialist:^a^				
Lack of evidence to support CRS and HIPEC	5 (3.9%)	3 (6.8%)	2 (2.4%)	.218
The morbidity and mortality of CRS and HIPEC is too high	5 (3.9%)	1 (2.3%)	4 (4.8%)	.490
I do not have access to an HIPEC specialist	13 (10.2%)	0	13 (15.5%)	.006
I refer patients	105 (82.0%)	39 (88.6%)	66 (78.6%)	.159
The NCCN guidelines	5 (3.9%)	2 (4.5%)	3 (3.6%)	.787
What indications have you used to refer patients for CRS and HIPEC?^a^				
Colon cancer	70 (54.7%)	29 (65.9%)	41 (48.8%)	.065
Gastric cancer	45 (35.2%)	16 (36.4%)	29 (34.5%)	.836
Peritoneal mesothelioma	40 (31.3%)	13 (29.5%)	27 (32.1%)	.763
High-grade appendiceal cancer	56 (43.8%)	27 (61.4%)	29 (34.5%)	.004
Ovarian cancer	3 (2.3%)	1 (2.3%)	2 (2.4%)	.969
I did not have patients to refer	1 (0.8%)	1 (2.3%)	0	.165
I do not refer patients	10 (7.8%)	2 (4.5%)	8 (9.5%)	.319
Low-grade appendiceal cancer (pseudomyxoma)	85 (66.4%)	31 (70.5%)	54 (64.3%)	.483
Peritoneal metastasis	4 (3.1%)	0	4 (4.8%)	.141
Other cancers	1 (0.8%)	0	1 (1.2%)	.467
Advanced cancer with metastasis	1 (0.8%)	0	1 (1.2%)	.467
What factors may influence your decision to refer in the future?^a^				
A change in the NCCN guidelines	37 (28.9%)	11 (25.0%)	26 (31.0%)	.480
A Phase III RCT confirming a survival advantage of CRS/HIPEC	27 (21.1%)	11 (25.0%)	16 (19.0%)	.433
Establishing a relationship with an HIPEC center or surgeon	34 (26.6%)	10 (22.7%)	24 (28.6%)	.907
I refer patients	66 (51.6%)	23 (52.3%)	43 (51.2%)	.477
For which cancers with peritoneal metastases would you consider CRS and HIPEC as a possible therapeutic option in appropriately selected cases?^a^				
Any cancer with peritoneal metastasis	58 (45.3%)	17 (38.6%)	41 (48.8%)	.272
Appendiceal cancer	88 (68.8%)	37 (84.1%)	51 (60.7%)	.007
Colon cancer	82 (64.1%)	31 (70.5%)	51 (60.7%)	.275
Gastric cancer	43 (33.6%)	18 (40.9%)	25 (29.8%)	.205
Ovarian cancer	80 (62.5%)	36 (81.8%)	44 (52.4%)	.001
Peritoneal mesothelioma	48 (37.5%)	25 (56.8%)	34 (40.5%)	
Please indicate the 5-year overall survival rate for patients undergoing CRS and HIPEC in an experienced center for Colon cancer with limited peritoneal spread:				
≤5%	1 (0.8%)	0	1 (1.2%)	
≤30%	28 (21.9%)	8 (18.2%)	20 (23.8%)	.804
30–50%	60 (46.9%)	22 (50.0%)	38 (45.2%)	
≥80%	28 (21.9%)	11 (25.0%)	17 (20.2%)	
Do not know	11 (8.6%)	3 (6.8%)	8 (9.5%)	
Please indicate the 5-year overall survival rate for patients undergoing CRS and HIPEC in an experienced center for peritoneal mesothelioma				
≤5%	7 (5.5%)	2 (4.5%)	5 (6.0%)	
≤30%	39 (30.5%)	14 (31.8%)	25 (29.8%)	
30–50%	45 (35.2%)	14 (31.8%)	31 (36.9%)	.647
≥80%	10 (7.8%)	2 (4.5%)	8 (9.5%)	
Do not know	27 (21.1%)	12 (27.3%)	15 (17.9%)	
Please indicate the 5-year overall survival rate for patients undergoing CRS and HIPEC in an experienced center for low-grade appendiceal neoplasm				
≤5%	1 (0.8%)	0	1 (1.2%)	
≤30%	15 (11.7%)	7 (15.9%)	8 (9.5%)	
30–50%	33 (25.8%)	6 (13.6%)	27 (32.1%)	.113
≥80%	67 (52.3%)	28 (63.6%)	39 (46.4%)	
Do not know	12 (9.4%)	3 (6.8%)	9 (10.7%)	
What is the 30-day mortality after CRS and HIPEC in a specialized center?				
0.005	16 (12.5%)	6 (13.6%)	10 (11.9%)	
0.1	24 (18.8%)	9 (20.5%)	15 (17.9%)	
0.2	12 (9.4%)	2 (4.5%)	10 (11.9%)	.631
≤2%	21 (16.4%)	9 (20.5%)	12 (14.3%)	
Do not know	55 (43.0%)	18 (40.9%)	37 (44.0%)	

Abbreviations: CRS- cytoreductive surgery; HIPEC- hyperthermic intraperitoneal chemotherapy; NCCN- National Comprehensive Cancer Network.

a multiple responses were allowed for this question.

**Fig. 1:**
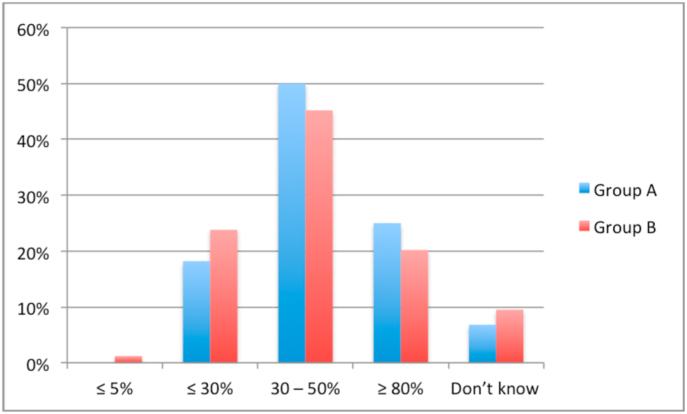
Respondents' knowledge regarding 5-year survival rate after CRS and HIPEC for Colon cancer with limited peritoneal spread.

**Fig. 2:**
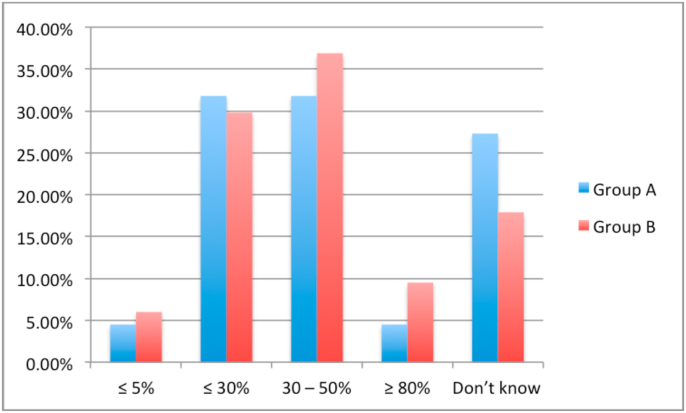
Respondents' knowledge regarding 5-year survival rate after CRS and HIPEC for peritoneal mesothelioma.

**Fig. 3:**
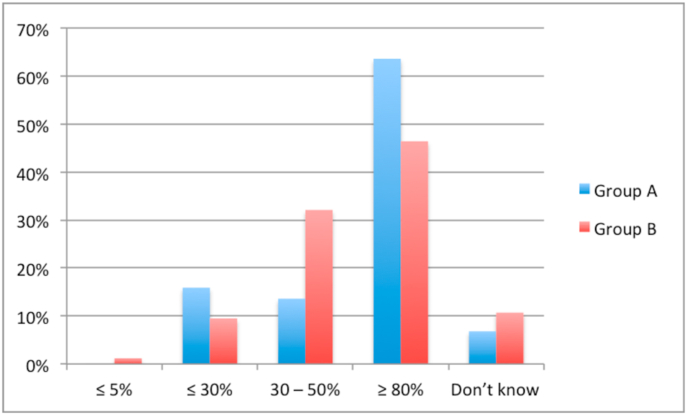
Respondents' knowledge regarding 5-year survival rate after CRS and HIPEC for low-grade appendiceal neoplasm.

**Fig. 4:**
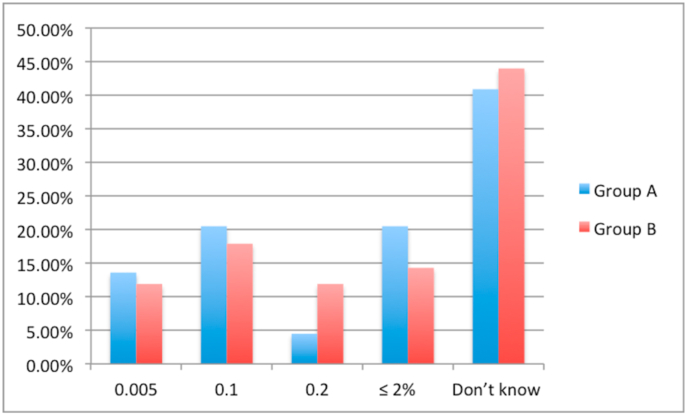
Respondents' knowledge regarding 30-day mortality after CRS and HIPEC.

A lack of access to HIPEC specialists was reported by 15.5% of respondents in group B and 0% in group A (*P* = .006). A similar proportion of respondents in group A (70.5%) and group B (70.2%) reported referring patients to a HIPEC specialist. Only 60.7% of respondents in group B cited HIPEC as a possible therapeutic option for appendiceal cancer compared to 84.1% of respondents in group A (*P* = .007). Similarly, only 52.4% of respondents in group B cited HIPEC as a treatment for ovarian cancer compared to 81.8% of respondents in group A (*P* = .001). Fig. 1, Fig. 2, Fig. 3, Fig. 4 show the respondents’ knowledge regarding the 5-year overall survival rate of patients undergoing CRS and HIPEC and the 30-day mortality rate after CRS and HIPEC in a specialized center, respectively.

## 3 Discussion

Our study assessed the exposure to the use of CRS and HIPEC for PC of surgeons in Riyadh, Saudi Arabia. The surgeons’ knowledge and background regarding the indications for CRS and HIPEC, exposure to and experience with CRS and HIPEC, and their access to a HIPEC specialist are important factors that reflect on the management of patients with PC. Few similar studies have been reported, and this is the first study of its type to focus on surgeons in Saudi Arabia. The Results of this study will be useful when developing new training guidelines regarding the use of CRS and HIPEC for eligible patients.

CRS and HIPEC are well-established therapies for appendiceal cancer with PC and result in a favorable long-term survival rate [11]. However, only 60.7% of respondents in group B and 84.1% of respondents in group A recognized that CRS and HIPEC can be used to treat appendiceal cancer. A similar study reported that 91% of general surgeons identified appendiceal cancer as a peritoneal malignancy suitable for HIPEC treatment [8]. Our Results were consistent with a study that reported that 51% of the respondents correctly identified appendiceal adenocarcinoma as an indication for CRS and HIPEC, 66% of the respondents identified high grade mucinous appendiceal cancer as an indication, and 68% of respondents reported low grade mucinous appendiceal cancer as an indication [12].

Stage III epithelial ovarian cancer has been reported to respond well to HIPEC and interval CRS, with a longer recurrence-free survival and overall survival than when surgery alone was used as treatment and no increase in the rates of adverse effects [13]. Based on this previous study, the National Comprehensive Cancer Network (NCCN) included HIPEC in their guidelines as a management option for interval debulking surgery (NCCN clinical practice guidelines Version 1.2019–March 8, 2019 OV-2) as mentioned by Stefano Cianci et al. [14,15] However, only 52.4% of the respondents in group B and 81.8% in group A correctly identified ovarian cancer as an indication for CRS and HIPEC.

In this study, 70.50% of respondents in group A and 60.7% in group B reported colorectal cancer carcinomatosis (CRC-C) as an indication for CRS and HIPEC; however, only 46% of respondents correctly identified CRC-C as an indication for CRS and HIPEC in a previous study, leading to the development of a referral checklist that includes a list of indications and contraindications for CRS and HIPEC [16]. The development of a referral checklist may be effective to provide patients with optimal care, as up to 90.9% of respondents in group A and 91.7% in group B reported treating patients with GI cancers, though only 54.5% of respondents in group A and 51.2% in group B were exposed to a HIPEC center during residency or fellowship training and only 31.8% of respondents in group A and 41.7% in group B learned about CRS and HIPEC during their training programs. As every surgical team bears the responsibility to minimize morbidity and mortality and provide patients with optimal management options, it is essential that general surgery training programs should include surgical oncology rotations, seminars, and online webinars that discuss the management of patients with advanced malignancies. Adequate training and access to HIPEC specialists are crucial for general surgeons who treat patients with cancer.

As the management of patients with PC is complex, strategies to improve communication between HIPEC and non-HIPEC centers are necessary, including a referral system or a monthly multidisciplinary HIPEC meeting that includes representatives from all Riyadh medical centers to discuss and refer eligible patients. As multidisciplinary meetings have been reported to effectively alter the diagnosis and management of a significant number of patients [7], this strategy may be useful in improving patient outcomes. The healthcare provided to patients with cancer should be equal at all medical centers.

This study is not without limitations. The response rate is relatively low, which may be due to the length of the survey or the lack of interest or knowledge in the topic. Also as this study was conducted only in Saudi Arabia, we believe it may not be applicable globally and further studies are needed.

In conclusion, the lack of knowledge and access to CRS and HIPEC centers are major obstacles to the proper care of patients with PC. Therefore, new strategies to increase the awareness and knowledge of CRS and HIPEC are necessary, and the implementation of a practical HIPEC center referral system is essential.

## Provenance and peer review

Not commissioned, externally peer-reviewed.

## Ethical Approval

This study was approved by the institutional review board committee at King Saud University Medical City

## Consent

Informed consent was obtained from all participants included in this study.

## Author contribution

All authors contributed to study design and conception, data collection, and interpretation.

Dr.Alshammari contributed to data analysis. Dr Alshamri, Dr. AlOtaibi and Dr. Alanazi wrote the first draft of the article. Dr. Bin Traiki, Dr Alnmry, Dr. Albdah, and Dr.Alhassan reviewed, critiqued, and edited the first draft, and approved the final version for publication.

## Registration of Research Studies

UIN: Researchregistry6829

## Guarantor

Dr. Wadha AlOtaibi.

## Declaration of competing interest

All authors disclose no financial or personal relationships with other people or organisations that could inappropriately influence their work.

## References

[1] Desai J.P., Moustarah F. (2020). Peritoneal metastasis. StatPearls. Treasure Island (FL).

[2] Franko J., Shi Q., Meyers J.P., Heinemann V., Falcone A., Tebbutt N.C. (2016). Prognostic value of isolated peritoneal versus other metastatic sites in colorectal cancer (CRC) patients treated by systemic chemotherapy: findings from 9,265 pts in the ARCAD database. J. Clin. Oncol..

[3] Helm J.H., Miura J.T., Glenn J.A., Marcus R.K., Larrieux G., Jayakrishnan T.T. (2015). Cytoreductive surgery and hyperthermic intraperitoneal chemotherapy for malignant peritoneal mesothelioma: a systematic review and meta-analysis. Ann. Surg Oncol..

[4] Jafari M.D., Halabi W.J., Stamos M.J., Nguyen V.Q., Carmichael J.C., Mills S.D. (2014). Surgical outcomes of hyperthermic intraperitoneal chemotherapy: analysis of the American College of Surgeons national surgical quality improvement program. JAMA Surg.

[5] Verwaal V.J., Bruin S., Boot H., van Slooten G., van Tinteren H. (2008). 8-year follow-up of randomized trial: cytoreduction and hyperthermic intraperitoneal chemotherapy versus systemic chemotherapy in patients with peritoneal carcinomatosis of colorectal cancer. Ann. Surg Oncol..

[6] Bernaiche T., Emery E., Bijelic L. (2018). Practice patterns, attitudes, and knowledge among physicians regarding cytoreductive surgery and HIPEC for patients with peritoneal metastases. Pleura Peritoneum.

[7] Basta Y.L., Bolle S., Fockens P., Tytgat K.M.A.J. (2017). The value of Multidisciplinary Team Meetings for Patients with gastrointestinal malignancies: a systematic review. Ann. Surg Oncol..

[8] Braam H.J., Boerma D., Wiezer M.J., van Ramshorst B. (2015). Cytoreductive surgery and HIPEC in treatment of colorectal peritoneal carcinomatosis: experiment or standard care? A survey among oncologic surgeons and medical oncologists. Int. J. Clin. Oncol..

[9] https://www.google.com/forms/about/.

[10] Agha R., Abdall-Razak A., Crossley E., Dowlut N., Iosifidis C., Mathew G., for the STROCSS Group (2019). The STROCSS 2019 guideline: strengthening the reporting of cohort studies in surgery. Int. J. Surg..

[11] Moaven O., Votanopoulos K.I., Shen P., Mansfield P., Bartlett D.L., Russell G. (2020). Health-related quality of life after cytoreductive surgery/HIPEC for mucinous appendiceal cancer: Results of a multicenter randomized trial comparing oxaliplatin and mitomycin. Ann. Surg Oncol..

[12] Siddiqui J., Brown K., Zahid A., Young C.J. (2020). Current practices and barriers to referral for cytoreductive surgery and HIPEC among colorectal surgeons: a binational survey. Eur. J. Surg. Oncol..

[13] van Driel W.J., Koole S.N., Sikorska K., van Leeuwen J.H.S., Schreuder H.W.R., Hermans R.H.M. (2018). Hyperthermic intraperitoneal chemotherapy in ovarian cancer. N. Engl. J. Med..

[14] Cianci S., Riemma G., Ronsini C., De Franciscis P., Torella M., Schiattarella A. (2020). Hyperthermic intraperitoneal chemotherapy (HIPEC) for ovarian cancer recurrence: systematic review and meta-analysis. Gland Surg..

[15] National Comprehensive Cancer Network (2020). Ovarian Cancer. https://www.nccn.org/professionals/physician_gls/pdf/ovarian.pdf.

[16] Spiegle G., Schmocker S., Huang H., Victor J.C., Law C., McCart J.A. (2013). Physicians' awareness of cytoreductive surgery and hyperthermic intraperitoneal chemotherapy for colorectal cancer carcinomatosis. Can. J. Surg..

